# Effect of Supercritical Carbon Dioxide Extraction Parameters on the Biological Activities and Metabolites Present in Extracts from *Arthrospira platensis*

**DOI:** 10.3390/md15060174

**Published:** 2017-06-12

**Authors:** Diego A. Esquivel-Hernández, José Rodríguez-Rodríguez, Sara P. Cuéllar-Bermúdez, J. Saúl García-Pérez, Elena I. Mancera-Andrade, Jade E. Núñez-Echevarría, Aura Ontiveros-Valencia, Magdalena Rostro-Alanis, Rebeca M. García-García, J. Antonio Torres, Wei Ning Chen, Roberto Parra-Saldívar

**Affiliations:** 1 Tecnologico de Monterrey, Escuela de Ingenieria y Ciencias, Campus Monterrey, Ave. Eugenio Garza Sada 2501, Monterrey, NL 64849, Mexico; diego.esquivel@itesm.mx (D.A.E.-H.); jrr@itesm.mx (J.R.-R.); garcia.saul@itesm.mx (J.S.G.-P.); ellen.mancera@gmail.com (E.I.M.-A.); aura_ontiveros@itesm.mx (A.O.-V.); magda.rostro@itesm.mx (M.R.-A.); rebeca.garcia.garcia@itesm.mx (R.M.G.-G.); 2 Laboratory of Aquatic Biology, KU Leuven Kulak, E. Sabbelaan 53, 8500 Kortrijk, Belgium; sara.cuellarbermudez@kuleuven.be; 3 Tecnologia Ambiental Biomex S.A. de C.V., Volcan Jorullo 5268, Zapopan, Jalisco 45070, Mexico; jadenunez89@gmail.com; 4 School of Chemical and Biomedical Engineering, Nanyang Technological University, 62 Nanyang Drive, Singapore 637457, Singapore; wnchen@ntu.edu.sg

**Keywords:** *Arthrospira platensis*, supercritical fluid extraction, antioxidants, antimicrobials, bioactive compounds, nutraceuticals, γ-linolenic acid, α-tocopherol, β-carotene, riboflavin

## Abstract

*Arthrospira platensis* was used to obtain functional extracts through supercritical carbon dioxide extraction (SFE-CO_2_). Pressure (P), temperature (T), co-solvent (CX), static extraction (SX), dispersant (Di) and dynamic extraction (DX) were evaluated as process parameters through a Plackett–Burman design. The maximum extract yield obtained was 7.48 ± 0.15% w/w. The maximum contents of bioactive metabolites in extracts were 0.69 ± 0.09 µg/g of riboflavin, 5.49 ± 0.10 µg/g of α-tocopherol, 524.46 ± 0.10 µg/g of β-carotene, 1.44 ± 0.10 µg/g of lutein and 32.11 ± 0.12 mg/g of fatty acids with 39.38% of palmitic acid, 20.63% of linoleic acid and 30.27% of γ-linolenic acid. *A. platensis* extracts had an antioxidant activity of 76.47 ± 0.71 µg GAE/g by Folin–Ciocalteu assay, 0.52 ± 0.02, 0.40 ± 0.01 and 1.47 ± 0.02 µmol TE/g by DPPH, FRAP and TEAC assays, respectively. These extracts showed antimicrobial activity against *Staphylococcus aureus* ATCC 25923, *Pseudomonas aeruginosa* ATCC 27853, *Escherichia coli* ATCC 25922 and *Candida albicans* ATCC 10231. Overall, co-solvent was the most significant factor for all measured effects (*p* < 0.05). *Arthrospira platensis* represents a sustainable source of bioactive compounds through SFE using the following extraction parameters P: 450 bar, CX: 11 g/min, SX: 15 min, DX: 25 min, T: 60 °C and Di: 35 g.

## 1. Introduction

In the last few years, we have seen a growing tendency to incorporate bioactive compounds from algae into traditional foods, cosmetics, and pharmaceutical products to replace components obtained by chemical synthesis [1]. *Arthrospira platensis* is a blue-green microalga (cyanobacteria) that grows extensively in tropical and subtropical water bodies, characterized by high levels of carbonate and bicarbonate. These cyanobacteria have been used since ancient times as a source of food, due to their high content of proteins (up to 70% dry weight), amino acids, fatty acids, vitamins, minerals, carbohydrates, phenolic compounds and pigments such as β-carotene and phycocyanins [2,3].

*A. platensis* has also been studied for its beneficial therapeutic effects. Several studies suggest that the consumption of raw *Arthrospira* or its extracts can prevent or inhibit cancer in humans and animals due to the chemopreventive properties of C-phycocyanin, β-carotene and the enzyme superoxide dismutase [4,5]. Other studies reported immuno-promoting effects due to increasing in the production of Interferon-γ [6,7], antimicrobial activity against *Staphylococcus aureus*, *Bacillus cereus*, *Escherichia coli*, and *Yersinia enterocolitica* from its extracts [8], and several pigments (e.g., β-carotene, zeaxanthin, chlorophyll and phycocyanin) that are related to its antioxidant activity [9,10].

For all those reasons, scientists show an increasing interest in the topic of extraction of natural antioxidants from cyanobacteria. For example, phenolic compounds are receiving attention mainly due to the wide range of their potential applications. These bioactive compounds retard or inhibit auto-oxidation by acting on radical scavengers and consequently are essential antioxidants that protect against the propagation of the oxidative chain [11]. In addition, it has several therapeutic properties (e.g., anticancer, antibacterial, and anti-inflammatory) [12].

Based on this information, we need deeper investigation about the extraction parameters and evaluation of bioactivities in extracts from *A. platensis*. Traditional techniques of extraction with organic solvents generate a significant amount of hazardous waste released to the environment and, in most cases, additional processes for treatment and disposal are required, raising the monetary and environmental costs of extraction methods [13].

Recently, new techniques for extraction and purification of bioactive compounds based on “greener extraction methods” have been receiving more attention due to their benefits, such as reduction in energy consumption, the use of alternative solvents (e.g., ethanol and carbon dioxide) and quality assurance of the extract/product. One of the most popular green technologies is the supercritical carbon dioxide extraction (SFE-CO_2_) [14,15]. SFE-CO_2_ is an extraction technique that uses the intrinsic properties of supercritical fluids with near liquid density, which allows them to have a liquid-like solvation. Their diffusivity, intermediate between liquid and gas, allows increased mass transfer between the extracted solute and the supercritical fluid [16]. CO_2_ is a suitable solvent for supercritical fluid extraction, since it is considered as an abundant resource obtained as a byproduct of various processes, such as fermentation, combustion, and ammonia synthesis, and it reaches supercritical state quickly (temperature of 304.12 K and pressure of 73.7 bar) [17].

Previously, SFE-CO_2_ has been used for the extraction of bioactive compounds from *Arthrospira platensis*, such as carotenoids [10], fatty acids [18], tocopherols [19], and phenolic compounds [8]. Data provided by these reports demonstrate the advantages of using SFE-CO_2_ since the developed processes were environmentally friendlier and faster than traditional solvent extraction. Moreover, these reports also support an important field of research to determine the reliability of the process parameters and the composition of the extracts. Although several studies have confirmed the bioactivity of *A. platensis* extracts (antimicrobial and antioxidant), data concerning how the process parameters affect those bioactivities are scarce. In this study, we examined the yield, the composition of *A. platensis* extracts and their bioactivities (antimicrobial and antioxidant) depending on the SFE-CO_2_ and process parameters. A Plackett–Burman design allows the selection of different factors of a process with the least possible number of treatments. In the case of SFE, this model has been exploited little [20], even though it provides a powerful tool for identifying the most important factors in the extraction of metabolites of *A. platensis* through SFE. The factors considered for this study were: pressure, temperature, co-solvent (ethanol), dispersant agent (glass pearls), and static and dynamic extraction. A literature review suggested that this is the first comprehensive study to determine the most significant factors and their values for the SFE for water-soluble vitamins. In addition, we examined the effect of the extraction factors on the content of carotenoids, tocopherols and fatty acids in *A. platensis* extracts and the antimicrobial activity against *Pseudomonas aeruginosa* ATCC 27853, *Staphylococcus aureus* ATCC 25923, *Candida albicans* ATCC 10231 and *Escherichia coli* ATCC 25922, as well as the antioxidant activity Folin–Ciocalteu, 1,1-diphenyl-2-picrylhydrazyl (DPPH), ferric reducing ability of plasma (FRAP), and Trolox equivalent antioxidant capacity (TEAC).

## 2. Results and Discussion

Several factors affect the SFE. The solubility of different compounds can be controlled mainly by the pressure and temperature of the SFE. Another important factor is the polarity of the solvents used due to the non-polar nature of the CO_2_ in a supercritical state. The use of polar and environmentally safe co-solvents such as ethanol is widely recommended to improve the extraction of polar compounds [21]. Furthermore, other factors allow higher extraction yields, such as the use of dynamic and static stages instead of a semi-continuous extraction process [22]. A dispersing agent, such as glass beads, helps to prevent the selective channeling of the CO_2_ [23]. Because of those considerations, six scanning process factors in SFE were evaluated in this study to determine which are the most significant for the extraction of bioactive metabolites and their antimicrobial and antioxidant activities. The treatments were chosen to cover a range of conditions considering the experimental limitations of the pilot plant scale, analytical equipment, and previous data reported for the extraction of bioactive metabolites from *A. platensis* [9,10,18,19,24,25,26].

### 2.1. Effect of Conditions of SFE on Extraction Yield in A. platensis Extracts

The extraction yield of the treatments performed was expressed as a percentage (w/w) (dw, dry weight) (Section 3.3 Equation (1)) and appears in Table 1. Table 1 contains the series of 12 treatments and their variations in CX, P, SX, DX, T and Di.

**Figure 1:**
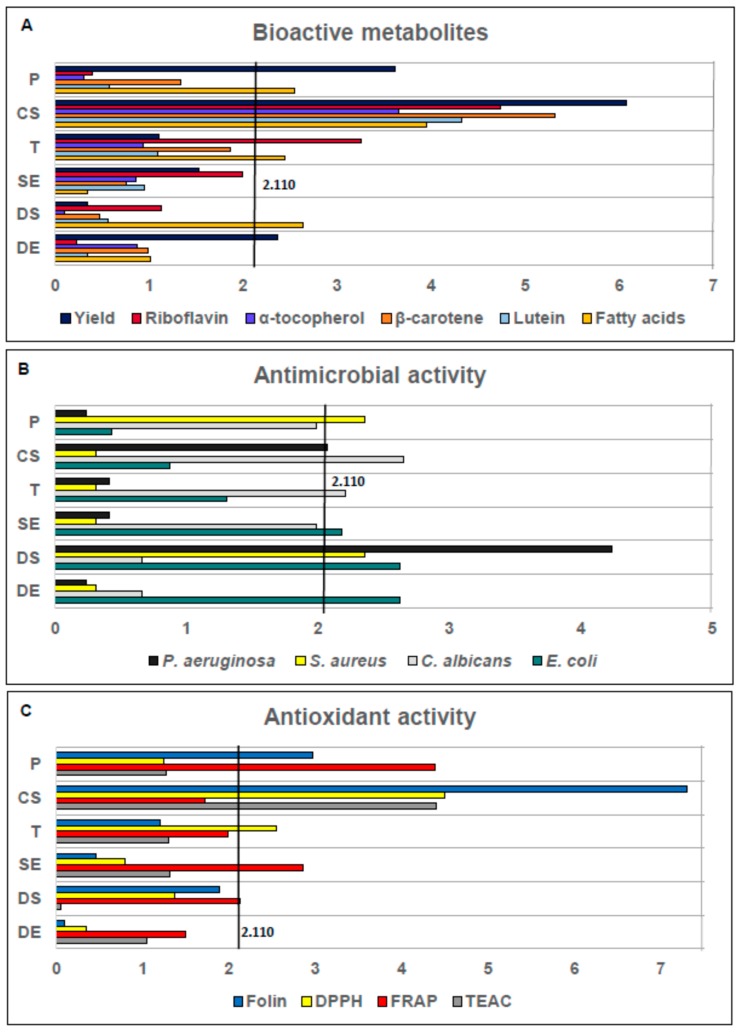
Standardized Pareto chart plots with the effect of each factor in the treatments, for: bioactive metabolites content (**A**); antimicrobial activity (**B**); and antioxidant activity (**C**). The vertical line in the chart tests the significance of the effects at 95% confidence level. (P) Pressure, (CX) Co-solvent, (T) Temperature, (SX) Static extraction, (Di) Dispersant, (DX) Dynamic extraction.

The highest extraction yield was seen in Treatment 8 (7.48 ± 0.15% w/w). Extraction at the highest levels of pressure, time and co-solvent gave maximum efficiency regarding total extraction yield. When working with supercritical conditions, the yield increases with the addition of co-solvent, because the extracted compounds have different polarities and, therefore the process becomes less selective [27]. For extraction yield, the statistical analysis showed that P, DX and CX Di were independently all significant factors (*p* < 0.05), (Figure 1A). The highest yield was obtained under the highest pressure, highest dynamic extraction and largest amount of co-solvent.

Temperature (T), SX and Di were not significant factors (*p* > 0.05). Overall, co-solvent was an essential element of SFE for total yield, riboflavin, α-tocopherol, β-carotene, lutein, and fatty acids (Figure 1A). In this study, the highest yield value is twelve times above the reported value (0.65% w/w) for *Arthrospira platensis* extracts without using co-solvent [10] and is similar (7.94% w/w) to extracts with co-solvent [25]. Therefore, it is evident that the use of co-solvent in SFE contributes to obtaining the highest yield in these extraction processes. Furthermore, the effect on the highest yield achieved with the highest pressure is similar to other reports in the literature [24,28]. Moreover, these results clearly prove the impact of the pretreatment of the biomass, since the samples were milled and selected with 1 mm (ps) sieve (Section 3.2). Therefore, the improvements in yield extraction can also be explained by the positive effect of a small particle size on the mass transfer of metabolites to SFE-CO_2_ [29].

### 2.2. Effect of Conditions of SFE on Water-Soluble Vitamins Content in A. platensis Extracts

**Figure 2:**
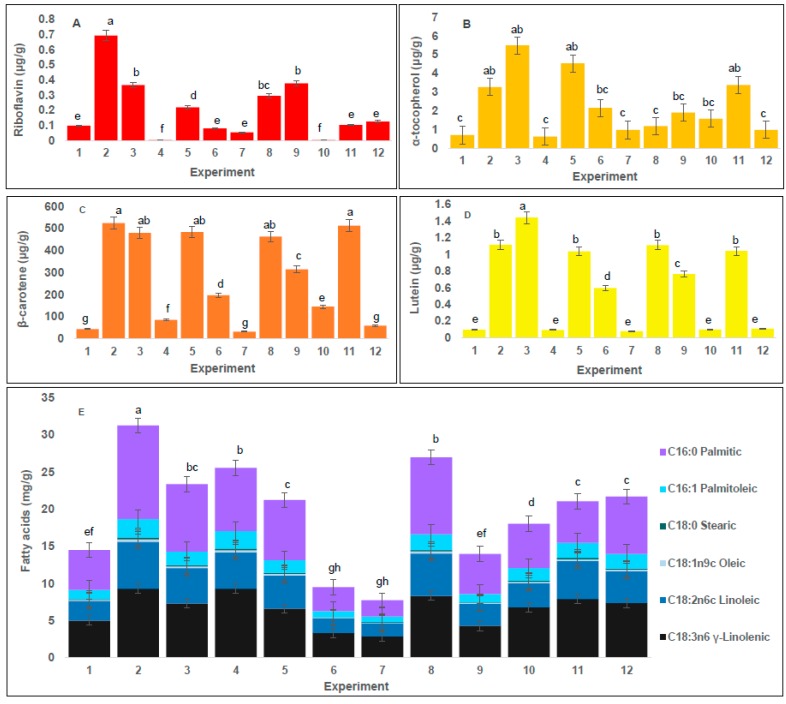
Bioactive metabolites content of SFE extracts of *Arthrospira platensis*: (**A**) Riboflavin (μg/g); (**B**) α-tocopherol (μg/g); (**C**) β-carotene (μg/g); (**D**) Lutein (μg/g); and (**E**) Fatty acids (mg/g). Values are presented as a mean ± standard deviation (*n* = 3). Bars with different letters correspond to values that are significantly different (Least Significant Difference LSD test, *p* < 0.05). All results are expressed in terms of percentage on dry basis (dw).

The content of water-soluble vitamins in microalgae and cyanobacteria is low, and found mixed with compounds of similar polarity. Thus, quantification typically requires a separate determination for each vitamin [30]. Furthermore, SFE primarily encourages removal of non-polar compounds due to the low polarity of CO_2_. To increase the range of metabolites that may be extracted, it is recommended to use polar co-solvents such as ethanol, and methanol, among others. Therefore the extracts obtained with SFE were partitioned with a mixture of ammonium acetate 10 mM and methanol as previously reported [31] for the simultaneous analysis of fat-soluble and water-soluble vitamins. In the case of vitamins, riboflavin was detected, but HPLC did not detect thiamine, niacin, and pyridoxine at the level of quantification (LOQ < 0.1 µg/g). Figure 2A shows the content of riboflavin obtained for all treatments.

The highest content of riboflavin (0.69 ± 0.09 µg/g) (dw) was achieved in Treatment 2. The extraction using high P, high T and short SX gave the highest efficiency regarding riboflavin content. According to the ANOVA, CX and T were independently all significant factors in this process (*p* < 0.05), (Figure 1A). The higher concentration of riboflavin in a shorter extraction time may be due to the possible oxidation of vitamins caused by a longer exposure to the oxygen present in the CO_2_ used in the extraction process [32]. Riboflavin is the least polar of the water-soluble vitamins [30], and extractions with SFE are more similar to non-polar solvent extractions. Our results represent a valuable finding to further research polar metabolite extraction using supercritical fluids and other co-solvents of higher polarity than ethanol.

### 2.3. Effect of Conditions of SFE on Tocopherol Content in A. platensis Extracts

Tocopherols are a diverse group of compounds; however, according to the results of mass spectrometer analysis, α-tocopherol was the only tocopherol detected (data not shown). Figure 2B lists the values of the concentration of tocopherols obtained for all SFE treatments. The highest concentration of tocopherols (5.49 ± 0.10 µg/g) (dw) was achieved in Treatment 3; furthermore, the CX was an independently significant factor (*p* < 0.05) (Figure 1A). Although the highest content of tocopherols obtained here was lower than previously reported [19], the authors only focused on the optimization of tocopherol content in the extracts without using co-solvent. Therefore, the lower content of tocopherols present in our work may be due to the levels of co-solvent in the process, the non-polar nature of tocopherols [33] and or dilution effect caused by extraction of other molecules with these parameters in SFE [24]. Additionally, in our treatments, we focused not only on tocopherols but also on the content of other metabolites present in the extracts.

### 2.4. Effect of Conditions of SFE on Carotenoids Content in A. platensis Extracts

According to the literature, the biomass of *Arthrospira* includes several carotenoids such as β-carotene, β-cryptoxanthin, zeaxanthin, and astaxanthin, among others [9,10,25]. Carotenoids are divided into two classifications: carotenes and xanthophylls. Carotenes were quantified by a β-carotene standard curve and xanthophylls were quantified by a lutein standard curve, since these two carotenoids are structurally representative of carotenes and xanthophylls, respectively, and they also have a great number of bioactivities reported [34]. Figure 2C,D shows the concentration of β-carotene and lutein, respectively, obtained for all SFE treatments. The highest content of β-carotene (524.46 ± 0.10 µg/g) (dw) was achieved in Treatment 2. However, in Treatment 11 with P 150 bar and SX 5 min, an important content of β-carotene was also obtained (513.06 ± 0.05 µg/g) (dw). These results may be due to a change in the solubility of carotenoids as a function of P and T [35]. For β-carotene content, CX emerged as an independently significant factor (*p* < 0.05), (Figure 1A). It can be concluded that CX has an important effect in extractions. The highest content of β-carotene reported in this study is lower than previously reported for *Arthrospira* [9], however, in that study, the extraction was performed for 100 min, while our results obtained similar concentrations in 30 min. Furthermore, β-carotene content in our treatments was higher than the results obtained in other reports with *A. platensis* [36], in which the extraction was done without co-solvent. Our data confirm the statistical significance of the co-solvent factor in the extraction process of β-carotene. For lutein, the highest content (1.44 ± 0.10 µg/g) (dw) was achieved in Treatment 3. Treatment 3 achieved the highest content for both lutein and α-tocopherol. Moreover, for lutein content, the use of co-solvent also emerged as an independently significant factor (*p* < 0.05), (Figure 1A). These results may be due to a change in the solubility of lutein as a function of temperature and pressure [37]. Lutein production from *Arthrospira* has been previously reported [25], but its concentration had not been determined by quantification with analytical standards. In addition, lutein content is very similar to results reported for extracts from the microalgae *Scenedesmus almeriensis,* a strain distinguished by its high level of lutein [38]. Therefore, these results offer an important finding for further investigation on the extraction conditions to improve lutein extraction from *S. almeriensis*.

### 2.5. Effect of Conditions of SFE on Fatty Acid Content in A. platensis Extracts

Due to the non-polar nature of SFE, fatty acids represented about 30% of the total compounds present in *Arthrospira* SFE extracts. Figure 2E shows the concentration of fatty acids obtained from all SFE treatments. The highest content of fatty acids (32.11 ± 0.12 mg/g) (dw) was achieved in Treatment 2. Extraction with high pressure, high temperature and shorter extraction time give the highest efficiency regarding fatty acid content. It is interesting to note that in Treatment 8, with longer time of extraction and without dispersant, considerable amounts of fatty acids were also obtained compared to other tested conditions. For fatty acid extraction and unlike the responses for the rest of the conditions tested, it was possible to observe the effect of dispersant agents in SFE since the same response was obtained with the use of dispersant and with longer extraction time. In addition, we found that the dispersant could be reused. Additionally, the use of a dispersing agent such as glass beads has been reported to be useful for preventing the selective channeling of the CO_2_ [39] and thus extraction of compounds by SFE is enhanced. Therefore, fatty acid extraction was facilitated by using glass beads, and for the highest yield of fatty acids, CX, P, T and Di were independently all significant factors (*p* < 0.05), (Figure 1A).

Overall, CX plays an important role in the extractions of all the metabolites analyzed (carotenoids, water-soluble vitamins, among others), and our results demonstrated that the addition of ethanol enhances the performance of the extraction process. The use of co-solvent improved extraction and solubility of the lipids due to molecular interactions between co-solvent and solute particles [40]. For example, a significant increase in the solubility of some fatty acids when ethanol was used as co-solvent has been reported, due to the interactions of hydrogen bonds [41]. The fatty acid profile was consistent with previous reports [36]; however, they reported about 3–5 times larger amounts of fatty acids compared to our study. This result can be explained by the use of longer extraction times. The composition of fatty acids in the Extract 2 was 39.38% of palmitic acid, 0.75% of stearic acid, 7.88% of palmitoleic acid, 1.06% of oleic acid, 20.63% of linoleic acid and 30.27% of γ-linolenic acid (total fatty acids, 32.11 ± 0.12 mg/g). These percentages are similar to those previously reported for *Arthrospira* [26], although the γ-linolenic acid content is slightly higher than previously reported [24,42,43]. In previous studies [18], a larger amount of palmitic acid (53.09%) and a smaller amount of γ-linolenic acid (15.8%) have been reported. However, in our study, we found different proportions, such as double the amount of γ-linolenic acid and a lower amount of palmitic acid, probably due to the effect of growth conditions on cell metabolites [44]. This could be of practical significance since γ-linolenic acid is considered a bioactive compound with proven effect on various mechanisms against inflammatory conditions such as dermatitis, diabetes, rheumatoid arthritis and premenstrual syndrome [18]. These treatments are promising for the extraction of γ-linolenic acid from a natural source with potential pharmaceutical applications.

### 2.6. Effect of Conditions of SFE on Antimicrobial Activity in A. platensis Extracts

Antimicrobial activity of *A. platensis* extracts was tested against four different microorganisms, including pathogenic Gram-positive bacteria *Staphylococcus aureus* (ATCC 25923), Gram-negative bacteria *Pseudomonas aeruginosa* (ATCC 27853), *Escherichia coli* (ATCC 25922) and one yeast strain *Candida albicans* (ATCC 10231) and the results are presented in Table 2. Most of the plates showed zones of growth inhibition; the diameters of these zones were measured in cm. Extract 4 was the most active against *S. aureus* and *P. aeruginosa* with 0.85 ± 0.19 cm of inhibition zone. According to the ANOVA, CX and Di were significant factors to facilitate the inhibition of *P. aeruginosa* while P and Di were independently significant factors for inhibiting *S. aureus* (*p* < 0.05), (Figure 1B). Extracts 7 and 10 were the most efficient against *C. albicans* with 0.90 ± 0.17 and 0.90 ± 0.22 cm of inhibition zones, respectively. CX and T were independently significant factors for *C. albicans* (*p* < 0.05), (Figure 1B). For *E. coli*, the most effective extract was Treatment 10 (similar to *C. albicans*) with 1.01 ± 0.06 cm of inhibition zone, with SX, Di and DX as the most independently significant factors to inhibit *E. coli* (*p* < 0.05), (Figure 1B).

Our results showed that when employing 4 g/min of co-solvent, the compounds responsible for the antimicrobial activity are selectively extracted. These data indicate that the best results regarding antimicrobial activity were obtained by lowering the amounts of co-solvent. Moreover, for *S. aureus, P. aeruginosa,* and *C. albicans* an extraction pressure equal to 150 bar seemed to be optimal to extract compounds with antimicrobial activity because higher pressure values resulted in less active extracts. However, for *E. coli*, the increase of extraction pressure produced extracts with higher antimicrobial activity. Specifically, the antimicrobial activity of *A. platensis* extracts can be explained by the presence of γ-linolenic acid [45]. Since the composition of fatty acids (Figure 2E) in the extract from Treatment 4 show an extraordinary amount of γ-linolenic acid, the antimicrobial activity seen matched the trend. However, for the other extracts with higher antimicrobial activity (Treatments 7 and 10) the composition of fatty acids (Figure 2E) is small, compared to Extract 4; therefore, the antimicrobial activity found in these extracts cannot be entirely attributed to γ-linolenic acid because the fatty acid analysis indicated the presence of various other fatty acids, specifically, palmitoleic and oleic acids with reported antimicrobial activities [46]. Hence, the antimicrobial activity found in *A. platensis* extracts could be linked to a synergy of all these fatty acids. Overall, Extract 4 represents a promising candidate for antimicrobial activity because it was effective against Gram-positive and Gram-negative. In addition, Extract 10 appears as a good candidate for antimicrobial activity because it was active against Gram-negative bacteria and yeast with the highest inhibition zones of all extracts. Furthermore, the activity against Gram-negative bacteria observed in extracts from *A. platensis* shows an interesting application for SFE extracts from cyanobacteria as a source of antimicrobials. Certainly in the last few years, the antimicrobial activities of organic solvents extracts from several cyanobacteria like *Nostoc* [47] *Synechocystis* [48], *S. maxima* [49] and *M. aeruginosa* [50] have been shown to be effective against both Gram-positive and Gram-negative bacteria. These reports are consistent with our results since *A. platensis* extracts prepared with different solvents had similar effects on both types of microorganisms.

### 2.7. Effect of Conditions of SFE on Antioxidant Activity in A. platensis Extracts

Four different methods were used to evaluate the effect of extraction conditions on antioxidant compounds. Table 3 synthesizes the results for the extracts obtained under various conditions of SFE at different levels of phenolic compounds and degrees of antioxidant activity using DPPH, FRAP, and TEAC methods.

The Folin–Ciocalteu method was used as a reference of total phenolics that are considered vital contributors to the antioxidant activity. The total amount of phenolic compounds in the *A. platensis* extracts is shown in Table 3. The highest content of phenolic compounds (76.47 ± 0.71 µg GAE/g, (Gallic acid equivalents)) (dw) was achieved in Treatment 8. This result is similar to the performance for yield (Section 2.1). When working with SFE conditions, the yield increases with the addition of co-solvent, meaning that the extracted compounds include various polarities, not only non-polar compounds but also medium polar compounds can be obtained and therefore, the process becomes less selective [27]. For the content of phenolic compounds, the statistical analysis shows that CX was an independently significant factor (*p* < 0.05). The highest concentration of phenolic compounds was obtained under the highest P, DX and, CX, (Figure 1C). Although the maximum content of phenolic compounds obtained in our study was lower than previously reported (43.2 mg GAE/g) [3], earlier work used different solvents and processes to extract the phenolic compounds. However, our results were comparable to data from a study that used solid phase extraction (SPE) (2.1 µg GAE/g) [51]. The content of phenolic compounds observed in our work may be due to the presence of ethanol as co-solvent in the process and the partition of crude extracts with polar solvents (Section 3.2) to separate and concentrate polar compounds. Moreover, the differences in total phenolics content can be explained due to several other influencing factors such as geographical origin, environmental, seasonal and physiological fluctuations [52]. In addition, the extraction process has an evident influence on the total phenolic content which is evident in our study and many others [53].

The DPPH assay can quantify both electron transfer (ET) reactions and transfer of hydrogen atoms (THA); and the antioxidant activity is quantified by measuring changes in absorbance that are related to the ability of antioxidants to reduce DPPH radical [54]. The antioxidant activity (µmol/g of trolox equivalents TE) by DPPH presented in the *A. platensis* extracts can be seen in Table 3. The highest antioxidant activity by DPPH (0.52 ± 0.01 µmol TE/g) (dw) was achieved in Treatments 2, 3 and 11. These results are similar to the results obtained for β-carotene response (Section 2.4). For DPPH antioxidant activity, P, CX, T, and SX were independently significant factors (*p* < 0.05), (Figure 1C). According to these results, the antioxidant activity by DPPH of *A. platensis* extracts was enhanced by P increase at high T. However, a rise in T at low P caused a loss of antioxidant capacity. The behavior of these *A. platensis* extracts has been previously reported [55] with the extraction of β-carotene by SFE from *Chlorella vulgaris.* The characterization of extracts showed that these two treatments obtained substantial amounts of β-carotene, lutein, and α-tocopherol (Figure 2B–D), which appeared to be the main antioxidant components in *A. platensis* extracts.

The FRAP assay is in the group of antioxidants assays that quantify the ET, specifically reducing the compound Fe(TPTZ)^3+^ that interacts with the antioxidants in a sample and produces an intense blue color. The assay is carried out under acidic conditions (pH 3.6), to maintain the solubility of iron. Because it only quantifies ET mechanisms, it is useful to distinguish the fundamental mechanisms in the same sample by comparison with other antioxidant assays [56].

The antioxidant activity (µmol TE/g) by FRAP presented in the *A. platensis* extracts is shown in Table 3. The highest antioxidant activity by FRAP (0.40 ± 0.01 µmol TE/g) (dw) was achieved in Treatments 5 and 9. These results are similar to those obtained for β-carotene, lutein and α-tocopherol responses (Section 2.3 and Section 2.4). For the antioxidant activity assessed by FRAP, SX was an independently significant factor (*p* < 0.05), (Figure 1C). The characterization of extracts showed that Treatments 5 and 9 have important amounts of β-carotene, lutein, and α-tocopherol (Figure 2B–D). Therefore, similar to the results obtained with DPPH assay, these compounds seemed to be the main antioxidant components in *A. platensis* extracts. However and for FRAP assay, only SX was a significant factor. This result can be expected because FRAP assay only measured antioxidant mechanisms that involves an ET mechanism, in contrast, DPPH measures ET and THA. Hence we hypothesize that static extraction affects the compositions of the extracts regarding the elements involved in ET mechanisms. In addition, we can suggest that antioxidants measured by DPPH are mostly the kind of compounds that use ET as an antioxidant mechanism.

The TEAC assay is based on the ability of antioxidants to capture the radical ABTS^+^, which is produced by oxidizing the compound ABTS. The product of this oxidation has an intense green color. The antioxidant capacity is measured by the ability of antioxidants present in the sample to decrease the color intensity by capturing the ABTS^+^ radical, causing a change in absorbance. This color is monitored at a wavelength of 734 nm. The TEAC assay quantifies both ET and THA mechanisms and can be performed over a wide range of pH [57].

The antioxidant activity (µmol TE/g) by TEAC presented in the *A. platensis* extracts can be seen in Table 3. The highest antioxidant activity by TEAC (1.67 ± 0.1 µmol TE/g) (dw) was achieved in Treatment 8. This result is similar to the performance for yield and phenolic compounds response (Section 2.1 and Section 2.7). For antioxidant activity assessed by TEAC, CX was an independently significant factor (*p* < 0.05), (Figure 1C). The characterization of extracts showed that this treatment has important amounts of β-carotene and lutein (Figure 2C,D). Both DPPH and FRAP assays produced similar results, and then we conclude that these compounds seemed to be the main antioxidant components in *A. platensis* extracts.

In general, Extracts 2 and 8 show the highest antioxidant activity with all the methods tested, and this result is in agreement with the composition of the extracts since Extract 2 shows the maximum content of β-carotene, lutein, and α-tocopherol. However, it is important to underline that it has been previously reported that besides β-carotene and lutein [58], cyanobacteria have other carotenoids such as zeaxanthin, astaxanthin, and echinenone that also show antioxidant activity against radicals. In addition, synergies between them probably contribute to the antioxidant activity of each *A. platensis* extract [59]. Moreover, since our extractions used ethanol as CX (significant factor for DPPH, TEAC, and Folin assays) we attained a better recovery of metabolites with different polarities. The effect of the co-solvent may be related to not only the change in polarity of the extraction fluid but also to its interaction with the matrix [59].

## 3. Materials and Methods

### 3.1. Chemicals

Carbon dioxide (Industrial grade) was obtained from Praxair S.A. (Guadalajara, Mexico) and ethanol from Reactivos Guadalajara (Guadalajara, Mexico). The α-tocopherol, thiamine, riboflavin, niacin, pyridoxine, ammonium acetate, acetic acid, fatty acid methyl esters mix (Supelco 37 component FAME Mix), Folin–Ciocalteu reagent, sodium carbonate, DPPH, sodium acetate, TPTZ, ferric chloride hexahydrate, trolox, and ABTS were obtained from Sigma-Aldrich (Toluca, Mexico). Lutein and β-carotene (analytical grade) were purchased from CaroteNature (Lupsingen, Switzerland). Methanol, acetone, hexane, sulfuric acid and water (HPLC grade) were acquired from Tedia (Monterrey, Mexico). Helium and nitrogen were purchased from Infra S.A. (Monterrey, Mexico). All reagents were ACS grade, unless otherwise stated.

### 3.2. Samples

All treatments were performed with biomass of *Arthrospira platensis* obtained from one batch culture of Tecnologia Ambiental Biomex (Guadalajara, Mexico). *Arthrospira* was cultivated in open ponds under greenhouses to avoid contamination using a modified Jourdan medium [24] (NaHCO_3_: 5.88 g /L, Na_3_PO_4_: 0.16 g/L, NaNO_3_: 0.92 g/L; MgSO_4_·7H_2_O: 7.07 g/L, FeSO_4_: 0.004 g/L and NaCl: 2 g/L) during 45 days, an agitation system with the propeller was used in the open ponds. The geographical location of the ponds was 20°14′0″ N, 103°35′0″ W. The biomass was air dried (60 °C, 24 h) to 10% RH, milled with a bar mill (22 bars 230 mm × 150 mm) (Biomex, Guadalajara, Mexico) for 10 h and sieved through 1 mm of particle size (ps). All samples were kept at 4 °C and protected from light.

### 3.3. Extraction Method

All extractions were carried out at the Biomex SFE pilot-scale plant (Guadalajara, Mexico) using a 100 mL extraction cell (Thar SFC SFE 100, Waters Corp., Milford, MA, USA) equipped with an automatic back pressure regulator (ABPR) controlling the extraction pressure, a CO_2_ pump (Thar Instruments Inc., Pittsburgh, PA, USA) and a co-solvent pump (Waters Corp.). The 17-4 PH stainless steel and Nitronic 60 extraction cell was equipped with 0.5 μm 17-4PH, Nitronic 60 and polyamide inlet and outlet filters. All extractions were performed with a 25 g/min CO_2_ flow. For each experiment, the extraction cell was filled with 35 g of cyanobacteria milled to 1 mm pore size. Extraction factors in this study were co-solvent, pressure, static extraction, dynamic extraction, temperature and 25 μm glass pearls as dispersant agent. In all experiments, dynamic extraction started after static extraction. Separator pressure and temperature were kept at 1 bar and 25 °C, respectively. All extractions used 96% aqueous ethanol as co-solvent with addition beginning after reaching the selected and continuing during extraction time. After each experiment, the extraction system (extraction cell, lines, and separator) were cleaned with CO_2_ and 10% aqueous ethanol. All extracts were concentrated in a rotary evaporator (IKA, Wilmington, DE, USA) and kept under N_2_ at −80 °C and protected from light. All the units are normalized to g of air dried cyanobacteria biomass. Average and standard deviation were determined for yield values estimated as follows (Equation (1)):

*Yield* = (*extracted solids* (g)/*initial biomass* (g)) × 100 (1)

### 3.4. Experimental Design

A Plackett–Burman design was used for the six experimental factors generating 12 experimental conditions tested with triplicates carried out in randomized run order. In this design, all six factors were tested at two different experimental levels: CX (g/min 4, 11); P (bar 150, 450); SX (min 5, 15); DX (min 25, 55); T (°C 40, 60); and, Di (g 0, 35). The response variables were extraction yield and the concentration of tocopherols, carotenoids, water-soluble vitamins (B-vitamins) and fatty acids. Antimicrobial activity against *P. aeruginosa*, *S. aureus*, *C. albicans* and *E. coli* and antioxidant activity through Folin–Ciocalteau, DPPH, FRAP and TEAC assays were also included. The results of each response variable and their statistical significance were analyzed with ANOVA (*p* > 0.05), using the statistical package Minitab 16 (State College, PA, USA).

### 3.5. Determination of Bioactive Metabolites in Samples

The scheme of the extraction procedure was based on previous reports with slight modifications while aiming the simultaneous extraction of water-soluble vitamins, carotenoids, tocopherols and fatty acids [31]. Briefly, concentrated samples of each treatment were first extracted with 3 mL of 10 mM ammonium acetate/methanol 50:50 (v/v). After shaking for 5 min, samples were placed in an ultrasound bath for 15 min. The bath temperature was controlled with ice to guarantee that the water temperature did not rise above 25 °C. The samples were centrifuged (Sorvall Legend XT, Thermo-Fisher, Waltham, MA, USA) at 4500 rpm for 15 min, and the supernatant was removed and placed in a volumetric flask. The solid residue of these extractions was re-extracted (2 times) as described above. The two extracts were placed in the same volumetric flask to reach a volume of 10 mL. This extract was named “polar extract” (PE). The solid residue after the two extractions was dissolved in 20 mL of hexane/acetone 80:20 (v/v) and this was named “non-polar extract” (NPE). For all determinations, 2 mL of PE (water-soluble vitamins) or NPE (carotenoids, tocopherols, and fatty acids) were filtered through a 0.20 μm nylon filter (Waters, Milford, MA, USA).

#### 3.5.1. Water-Soluble Vitamins Analysis (HPLC-PDA-FLD)

Water-soluble vitamins (thiamine, riboflavin, niacin, and pyridoxine) were quantified using an HPLC (Model 1200, Agilent Technologies, Inc., Santa Clara, CA, USA) equipped with autosampler, photodiode array detector (PDA), fluorescence detector (FLD), and a Zorbax Eclipse XDB C18 column (5 μm, 150 × 4.6 mm). The HPLC method based on previous work [31,60,61] used 10 mM ammonium acetate solution (pH 4.5) as phase A, methanol with 0.1% (v/v) acetic acid as phase B, and methanol with 0.3% (v/v) acetic acid as phase C. The mobile phase gradient consisted in 0/97, 3/97, 4/90, 6/90, 7/83, 9/83, 10/50, 13/50, 17/97 (min/%phase A) and 10/50, 13/50 (min/%phase C). The flow rate was 1 mL/min and the injection volume was 25 μL. Chromatograms were obtained at 245 nm for thiamine, 450 nm for riboflavin, 290 nm for niacin and 275 nm for pyridoxine, and 280 and 390 nm excitation and emission wavelength for pyridoxine; 280 and 530 nm excitation and emission wavelength for riboflavin. Quantification of water-soluble vitamins was performed with an external standard of thiamine, riboflavin, niacin, and pyridoxine.

#### 3.5.2. Carotenoid Analysis (HPLC-PDA)

Carotenoids were determined as in previous work [60] using an HPLC (Model 1200, Agilent Technologies, Inc.) equipped with an autosampler, a photodiode array detector (PDA), precolumn AJO 4348, and a Luna Silica 100 Å column (3μm, 150 × 4.6 mm) from Phenomenex Inc. (Torrance, CA, USA). Hexane/acetone 82:18 v/v at 1.2 mL/min in isocratic mode for 10 min was used as mobile phase and the injection volume was 25 μL. Chromatograms were obtained at 450 nm for β-carotene and lutein quantified using β-carotene and lutein standards.

#### 3.5.3. Tocopherol Analysis (GC-MS)

Tocopherols were analyzed by GC (Model 6890N, Agilent Technologies Inc.) equipped with an HP-5MS capillary column (30 m, 0.25 mm i.d., 0.25 μm film thickness) and a 5973N mass spectrometer with an electron ionization system set at 70 eV as a detector [24]. The carrier gas was helium at a flow rate of 0.8 mL/min. The column temperature was kept initially at 190 °C for 1 min, then gradually increased to 300 °C at 15 °C/min, and finally kept at 300 °C for 25 min. Extract aliquots (1 μL) were injected automatically with a 20:1 split mode. Injector and detector temperatures were set at 270 and 230 °C, respectively. Tocopherols were quantified based using a standard curve of α-tocopherol.

#### 3.5.4. Fatty Acid Analysis (GC-FID)

Fatty acids were determined through derivatization to fatty acid methyl esters (FAMEs) as previously described [24,60,62]. Briefly, samples were mixed with 400 μL of the internal standard (undecanoic acid 1000 mg/L in hexane/acetone 80:20 v/v) and 2 mL of methanol/sulfuric acid (93:7 v/v). These mixtures were maintained for 60 min at 80 °C and then cooled to room temperature. After addition of 5 mL of hexane, they were mixed in a vortex for 1 min and then set aside to allow phase separation. The organic phase was transferred into a 10-mL volumetric flask. Re-extraction of the polar phase was repeated 3–4 times until obtaining a 10 mL volume. FAMEs were analyzed with an Agilent 6890N GC (Agilent Technologies, Santa Clara, CA, USA), equipped with an SP2380 capillary column (30 m, 0.25 mm i.d., 0.20 μm film thickness) and a flame ionization detector (Model 19244-80560). The carrier gas was nitrogen at a flow rate of 0.8 mL/min. The column temperature was kept initially at 50 °C for 2 min, then gradually increased to 240 °C at 4 °C/min, and finally kept at 240 °C for 1 min. Extract aliquots (1 μL) were injected automatically with 20:1 in split mode. Injector and detector temperatures were set at 260 and 280 °C, respectively. Quantification of fatty acids was performed with an external standard of FAME mix (Supelco 37 component FAME Mix, Sigma).

### 3.6. Determination of Biological Activities in Samples

#### 3.6.1. Antimicrobial Activity

##### Test Microorganisms

The species employed included pathogenic Gram-positive bacteria (*Staphylococcus aureus*, ATTC 25923), Gram-negative bacteria (*Pseudomonas aeruginosa*, ATTC 27853; *Escherichia coli*, ATTC 25922) and one yeast strain (*Candida albicans*, ATTC 10231). All bacteria were inoculated in nutrient broth (BD, Cuautitlan Izcalli, Mexico) and incubated for 24 h at 37 °C, while *C. albicans* was inoculated on malt extract broth and incubated 24 h at 25 °C. Autoclaved Mueller Hinton agar (15 mL, BD) was dispensed into 100 × 15 mm sterile petri dishes (Corning, Monterrey, Mexico) and allowed to solidify under aseptic conditions. For each tested microorganism, an inoculum of 150 µL was adjusted to approximately 1 × 10^7^ cells/mL using the McFarland counting method. The inoculum was then applied to the surface of solid medium plates using a sterile swab. The plates were incubated at 37 °C (bacterial strains) and 25 °C (*C. albicans*) for 1 h.

##### Antimicrobial Testing

The antimicrobial activity of *A. platensis* SFE extracts was determined by the paper disk diffusion method [60]. Briefly, sterile, 4 mm diameter filter paper disks (Whatman # 1, Sigma-Aldrich Corp., St. Louis, MO, USA) were impregnated with 35 µL of *A. platensis* extracts or 35 µL of extracting solvent as negative control. Disks were air dried in a biological safety cabinet (Logic + Class II, Labconco, Kansas, MO, USA) removing solvent to an undetectable level. Agar plates previously inoculated with each test organisms were incubated for 1 h before placing extract impregnated paper disks on the plates which were then incubated at 37 °C for 24 h for bacteria plates while yeast plates were incubated at 25 °C for 72 h. After incubation, the diameters of the growth inhibition zones were measured in centimeters. All tests were performed in triplicate under sterile conditions. Discs impregnated with 35 µL of ampicillin and nystatin (10 µg/mL) were used as positive controls.

#### 3.6.2. Antioxidant Activity

##### Determination of Total Phenolics Folin–Ciocalteu Assay

The total phenolic content of *A. platensis* extracts was assessed according to the Folin–Ciocalteu method [63]. Briefly, 50 µL of *A. platensis* extract, 50 µL of Folin–Ciocalteu’s reagent, and 650 µL of distilled water were mixed. The solution was incubated for 5 min under darkness and at room temperature before adding 250 µL of 0.5 M Na_2_CO_3_. The solution was mixed and incubated for 2 h at 37 °C under darkness. Sample absorbance was measured at 765 nm on UV/VIS spectrophotometer (Model DR5000, Hach Co., Loveland, CO, USA). Gallic acid was used for the standard calibration curve.

##### DPPH Free Radical Scavenging Assay

This assay was a modification of previously reported work [60,64]. *A. platensis* extract aliquots (100 µL) were air dried and resuspended in 100 µL of methanol. DPPH solution in methanol (3 mL, 60 µM) was placed in a 15-mL centrifuge tube, and 75 µL of sample extract were added. Methanol was used as blank with DPPH methanol solution as a reference sample and Trolox as standard. The absorbance of the reaction mixture was measured at 517 nm on UV/VIS spectrophotometer (Model DR5000, Hach Co., Loveland, CO, USA).

##### FRAP Assay

This assay was based on previously reported work [65] with some modifications [60]. *A. platensis* extract aliquots (100 µL) were air dried and resuspended in 100 µL of methanol. The stock solutions included 300 mM acetate buffer (3.1 g C_2_H_3_NaO_2_·3H_2_O and 16 mL CH_3_OH), pH 3.6, 10 mM TPTZ (2,4,6-tripyridyl-s-triazine) solution in 40 mM HCl, and 20 mM FeCl_3_·6H_2_O solution. The fresh FRAP working solution was prepared by mixing 25 mL acetate buffer, 2.5 mL TPTZ solution, and 2.5 mL FeCl_3_·6H_2_O solution and then warmed to 37 °C before using. *A. platensis* extracts (100 μL) were allowed to react with 2900 μL of the FRAP solution for 30 min in dark conditions. Methanol was used as blank with FRAP solution as a reference sample and Trolox as standard. The absorbance of this reaction mixture (ferrous tripyridyl triazine complex) was measured at 593 nm on UV/VIS spectrophotometer (Model DR5000, Hach Co., Loveland, CO, USA).

##### TEAC Assay

This assay is based on the antioxidant ability to capture the 2,2’-azino-bis (3-ethylbenzthiazoline-6-sulphonic acid) radical. The procedures followed in this study are based on previously reported work [60,66]. *A. platensis* extract aliquots (100 µL) were air dried and resuspended in 100 µL of methanol. A working ABTS^+^ solution was prepared by mixing in equal quantities stock solutions of 7.4 mM ABTS^+^ and 2.6 mM potassium persulfate and allowing the mixture to react for 12 h at room temperature in the dark. A 1 mL of the working ABTS^+^ solution was then diluted by mixing with 60 mL methanol yielding an absorbance of 1.1 ± 0.02 units at 734 nm. A fresh working ABTS^+^ solution was prepared for each assay. *A. platensis* extracts (10 μL) were allowed to react for 2 h in the dark with 990 μL of the working ABTS^+^ solution. Methanol was used as blank with the working ABTS^+^ solution as a reference sample and Trolox as standard. The absorbance of this reaction mixture was measured at 734 nm on UV/VIS spectrophotometer (Model DR5000, Hach CO., Loveland, CO, USA).

## 4. Conclusions

In this work, we studied the effects of six factors (i.e., P, T, CX, SX, Di, and DX) of SFE from *A. platensis* on the following: total extraction yield, water-soluble vitamins, tocopherols, carotenoids, fatty acids, and antimicrobial and antioxidant activities. The Plackett–Burman design allowed finding the most significant factors and their responses for each metabolite, pathogenic microorganism, and antioxidant assay. This research is presented as a preliminary work before optimizing the extraction process of these metabolites and their bioactivities. SFE extracts from *A. platensis* presented important quantities of bioactive compounds. The highest yield extraction obtained was (7.48 ± 0.15% w/w) in Treatment 8 at P: 450 bar, CX: 11 g/min, SX: 5 min, DX: 55 min, T: 40 °C and Di: 0 g.

The highest content of bioactive metabolites such as β-carotene, lutein, tocopherols and γ-linoleic acid was obtained in Treatment 2 at P: 450 bar, CX 11/g/min, SX: 15 min, DX: 25 min, T: 60 °C and Di: 35 g. In addition, this treatment offers the maximum level of antioxidants and important level of antimicrobial activity against *S. aureus* ATCC 25923, *P. aeruginosa* ATCC 27853, *E. coli* ATCC 25922 and *C. albicans* ATCC 10231.

In general, CX (i.e., ethanol) was the most significant factor in all responses because it increases the polarity of CO_2_ and allows the extraction of larger amounts of the compounds. Moreover, ethanol and CO_2_ offer additional advantages such as being inexpensive and environmentally friendly solvents and potentially be reused for the case of carbon dioxide. Overall, we demonstrated that SFE CO_2_ was successful for *A. platensis* and its bioactive compounds.

## Figures and Tables

**Table 1:** Experimental matrix design for SFE conditions and extraction yields of *A. platensis* extracts ^1^.

Treatment	CX (g/min)	P (Bar)	SX (min)	DX (min)	T (°C)	Di (g)	Yield ^2^ (%)
1	4	450	5	25	40	35	1.90 ± 0.13 ^h^
2	11	450	15	25	60	35	5.59 ± 0.09 ^c^
3	11	450	5	55	60	0	6.85 ± 0.16 ^b^
4	4	150	5	55	60	35	2.02 ± 0.07 ^h^
5	11	150	15	55	40	35	5.62 ± 0.11 ^c^
6	4	150	15	55	60	0	2.34 ± 0.15 ^g^
7	4	150	5	25	40	0	1.52 ± 0.08 ^l^
8	11	450	5	55	40	0	7.48 ± 0.15 ^a^
9	11	150	15	25	40	0	3.71 ± 0.09 ^e^
10	4	450	15	55	40	35	2.48 ± 0.10 ^g^
11	11	150	5	25	60	35	4.78 ± 0.14 ^d^
12	4	450	15	25	60	0	3.01 ± 0.11 ^f^

^1^ Co-solvent (CX), pressure (P), static extraction (SX), dynamic extraction (DX), temperature (T), dispersant (Di). ^2^ Yield (w/w, dw) are represented as a mean ± standard deviation values (*n* = 3). Different letters indicate significant differences (Least Significant Difference LSD test, *p* < 0.05).

**Table 2:** Antibacterial activity of *Arthrospira platensis* extracts.

	Diameter of Effective Zone of Inhibition (cm) ^1^			
Treatment	*S. aureus*	*P. aeruginosa*	*C. albicans*	*E. coli*
1	0.75 ± 0.21 ^b^	0.65 ± 0.07 ^c^	0.80 ± 0.01 ^b^	0.65 ± 0.07 ^d^
2	0.75 ± 0.07 ^b^	0.65 ± 0.07 ^c^	0.85 ± 0.06 ^a^	0.70 ± 0.02 ^d^
3	0.65 ± 0.05 ^d^	0.75 ± 0.04 ^a^	0.65 ± 0.01 ^d^	0.65 ± 0.05 ^d^
4	0.85 ± 0.19 ^a^	0.70 ± 0.03 ^b^	0.65 ± 0.08 ^d,e^	0.75 ± 0.04 ^c,d^
5	0.60 ± 0.01 ^e^	0.60 ± 0.09 ^c^	0.70 ± 0.02 ^c^	0.80 ± 0.01 ^b^
6	0.70 ± 0.02 ^c^	--	0.70 ± 0.01 ^c^	0.75 ± 0.12 ^b,c^
7	0.70 ± 0.01 ^c^	--	0.90 ± 0.17 ^a^	0.65 ± 0.08 ^d^
8	--	--	0.60 ± 0.03 ^e^	0.65 ± 0.05 ^d^
9	0.75 ± 0.03 ^b^	0.60 ± 0.11 ^c^	0.65 ± 0.08 ^d,e^	0.70 ± 0.04 ^d^
10	0.65 ± 0.05 ^d^	0.65 ± 0.09 ^c^	0.90 ± 0.22 ^a^	1.01 ± 0.06 ^a^
11	0.70 ± 0.14 ^c^	0.65 ± 0.04 ^c^	--	0.70 ± 0.07 ^b,c^
12	--	--	0.70 ± 0.15 ^c^	0.60 ± 0.08 ^d^
Control (+)	1.10 ± 0.01	1.08 ± 0.05	1.23 ± 0.06	1.05 ± 0.01
Control (−)	--	--	--	--

^1^ Values are represented as a mean ± standard deviation (*n* = 3). Values with different superscript letters in the same column are significantly different (Least Significant Difference LSD test, *p* < 0.05).

**Table 3:** Antioxidant activity of *Arthrospira platensis* extracts ^1^.

	Folin–Ciocalteau	DPPH		FRAP	TEAC
Treatment	μg GAE/g	% Inhibition	μmol TE/g	μmol TE/g	μmol TE/g
1	23.22 ± 0.27 ^g^	4.04 ± 0.16 ^e^	0.23 ± 0.01 ^e^	0.31 ± 0.00 ^e^	0.56 ± 0.01 ^i^
2	55.40 ± 0.54 ^b^	11.05 ± 1.03 ^a^	0.52 ± 0.02 ^a^	0.34 ± 0.01 ^c,d^	1.12 ± 0.02 ^d^
3	36.74 ± 0.45 ^e^	11.04 ± 0.93 ^a^	0.52 ± 0.01 ^a^	0.33 ± 0.01 ^c,d^	0.58 ± 0.00 ^h^
4	17.96 ± 0.27 ^i^	4.16 ± 0.08 ^e^	0.24 ± 0.01 ^e^	0.17 ± 0.02 ^f^	0.89 ± 0.01 ^e^
5	48.83 ± 0.76 ^d^	5.97 ± 1.97 ^d^	0.31 ± 0.00 ^d^	0.40 ± 0.01 ^a^	0.91 ± 0.01 ^e^
6	22.57 ± 0.17 ^h^	4.08 ± 0.50 ^e^	0.23 ± 0.01 ^e^	0.39 ± 0.00 ^a^	0.51 ± 0.01 ^j^
7	17.72 ± 0.32 ^i^	4.01 ± 0.04 ^e^	0.22 ± 0.01 ^e^	0.13 ± 0.03 ^g^	0.87 ± 0.01 ^f^
8	76.47 ± 0.71 ^a^	10.23 ± 0.46 ^b^	0.40 ± 0.01 ^b^	0.11 ± 0.01 ^g^	1.67 ± 0.01 ^a^
9	53.12 ± 0.61 ^c^	1.01 ± 0.16 ^f^	0.13 ± 0.00 ^f^	0.40 ± 0.01 ^a^	1.37 ± 0.02 ^c^
10	9.51 ± 0.29 ^j^	4.07 ± 1.04 ^e^	0.23 ± 0.01 ^e^	0.37 ± 0.00 ^b^	0.65 ± 0.01 ^g^
11	38.85 ± 1.01 ^e^	10.99 ± 0.64 ^a^	0.51 ± 0.02 ^a^	0.39 ± 0.01 ^a^	1.47 ± 0.02 ^b^
12	28.38 ± 0.56 ^f^	4.99 ± 1.04 ^c^	0.27 ± 0.01 ^c^	0.35 ± 0.01 ^c^	0.55 ± 0.01 ^i^

^1^ Values are represented as a mean ± standard deviation (*n* = 3) w/w (dw). GAE: Gallic acid equivalents; TE: Trolox equivalents. Values in the same column with different superscript letters are significantly different (Least Significant Difference LSD test, *p* < 0.05).

## Abbreviations

RH	Relative humidity
ps	Particle size
P	Pressure
CX	Co-solvent (ethanol)
SX	Static extraction
DX	Dynamic extraction
T	Temperature
Di	Dispersant
dw	Dry weight
PE	Polar extract
NPE	Non-polar extract
PDA	Photodiode array detector
FLD	Fluorescence detector
MS	Mass detector
FID	Flame ionization detector
SFE	Supercritical fluid extraction
LOQ	Limit of quantification
GAE	Gallic acid equivalents
DPPH	1,1-diphenyl-2-picrylhydrazyl
FRAP	Ferric reducing ability of plasma
TEAC	Trolox equivalent antioxidant capacity
TE	Trolox equivalents
ABTS	2,2′-azino-bis(3-ethylbenzthiazoline-6-sulphonic acid)
